# An Unusual Cervical Tumor as Presentation of a Non-Hodgkin Lymphoma

**DOI:** 10.1155/2014/549619

**Published:** 2014-03-24

**Authors:** Tom De Greve, Lieve Vanwalleghem, Achiel Van Hoof, Kenneth Coenegrachts, Philippe Van Trappen

**Affiliations:** ^1^Obstetrics and Gynecology Department, AZ Sint-Jan Hospital, Ruddershove 10, 8000 Bruges, Belgium; ^2^Pathology Department, AZ Sint-Jan Hospital, Bruges, Belgium; ^3^Department of Hematology, AZ Sint-Jan Hospital, Bruges, Belgium; ^4^Radiology Department, AZ Sint-Jan Hospital, Bruges, Belgium

## Abstract

Rare cervical cancers are responsible for a minority of cases encountered by a clinician. However, behavioral patterns, management, and prognosis of certain rare cervical cancers differ from either squamous carcinomas or adenocarcinomas. Here we present a case of a locally advanced cervical tumor as a presentation of an extranodal cervical non-Hodgkin lymphoma (NHL), with a review of the current literature.

## 1. Introduction

Cervical cancers, as well as their precursors, originate from either squamous or glandular cell lineage. Most cases of cervical cancer are therefore either squamous cell carcinomas or adenocarcinomas. Rare cervical malignancies include tumors such as adenosquamous carcinoma (3.0%), clear-cell carcinoma (1.1%), neuroendocrine carcinoma (0.6%), lymphoma (0.3%), leiomyosarcoma (0.2%), and lymphoepithelioma-like carcinoma (0.1%) [1].

Behavioral patterns, management, and prognosis of certain rare cervical cancers are different from either squamous or adenocarcinomas. Here we present a rare cervical tumor, extranodal cervical NHL, with an unusual clinical and radiological presentation.

## 2. Case

A 75-year-old woman presented herself in our clinic with complaints of postmenopausal vaginal blood loss for a period of two weeks, bladder pressure, and a slight pain located at the right groin. Her past medical history consists of B-cell follicular NHL 18 years ago with right groin and thoracic lymph node involvement (stage IIIa). Treatment consisted of chemo- and radiation therapy, with no relapse until present.

**Figure 1:**
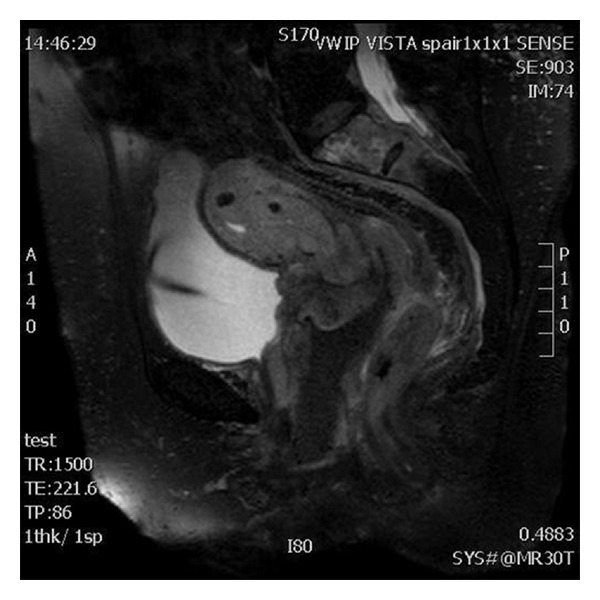
3D VISTA T2-weighted sagittal MRI images of the pelvis. Images show a locally invasive tumor, with heterogeneous rather high intensity signal, most likely originating from the cervix with transmural invasion of the uterus. Invasion into the anterior part of the vagina, bladder, lumbosacral plexus, sacrum, and mesorectum. (The vagina, low/dark signal, was filled with a mixture of barium and gel at the start of the MRI examination for accurate evaluation of vaginal wall.)

An irregular tumor mass of the cervix was visualized during gynecological examination. The surface was smooth with no exophytic or erosive components. The cervix cytology smear was normal. The bulky locally advanced cervical tumor extended in the right paracervical and parametrial tissue, fixating to the right pelvic rim, invading the posterior bladder wall and proximal 2/3 of the vaginal submucosa. No enlarged inguinal or supra clavicular lymph nodes were observed. On ultrasound examination a 4-by-4.2-centimeter highly vascularized cervical tumor extending into the right parametrium and bladder without mucosal infiltration was visualized. The right ureter seemed to be encased by the tumor causing hydroureteronephrosis. A magnetic resonance imaging (MRI) was performed showing a locally invasive tumor, with heterogenous high intensity signaling on T2-weighted images, most likely originating from the cervix with transmural invasion of the uterus. Invasion into the anterior part of the vagina, bladder, right pelvis, lumbosacral plexus, sacrum, and mesorectum was visualized (Figure 1). Bilateral hydroureteronephrosis was visualized.

The computed tomography (CT) scan showed a similar extent of cervix tumor. No enlarged lymph nodes or lesion in liver, lung, or bone was visualized.

A cystoscopy revealed no mucosal invasion. And multiple biopsies of the cervix and vagina were taken. The clinical diagnosis of possibly FIGO-stage IIIb cervical carcinoma was made.

**Figure 2:**
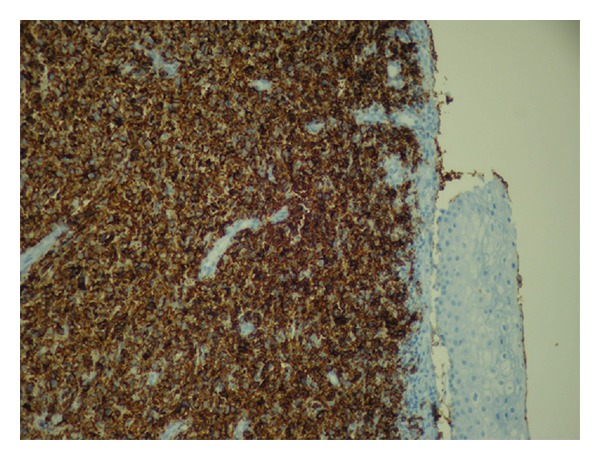
Tumor histology with positive CD20 immunohistochemical staining (staining the cell membrane), indicative for B-cell lymphoma.

Surprisingly, the biopsies revealed an NHL (Figure 2). Neoplastic cells were positive for CD20 and Ki67 100% and negative for CD3, CD5, CD10, BCL2, S100, and Cytokeratin. These findings confirmed the diagnosis of a diffuse high grade large cell B-cell NHL relapse presenting itself as a cervical tumor.

**Figure 3:**
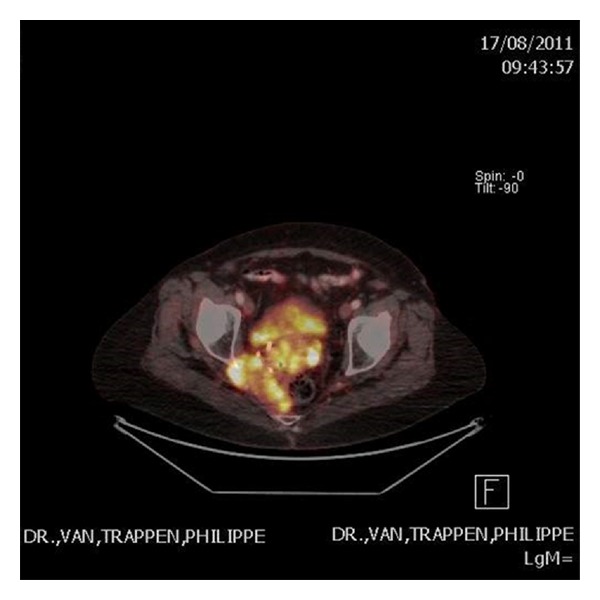
PET-CT visualizing FDG accumulation in the extranodal tumor located in the pelvis with tumor extension into the sacrum and the gluteal/obturator muscle on the right side.

A positron emission tomography- (PET-) CT showed fluorodeoxyglucose-18 (FDG) accumulation in the pelvic tumor with extension into the bony pelvis left, sacrum and the gluteal/obturator muscle on the right side (Figure 3). There was no evidence of other PET-positive lesions. The patient was staged as IE according to the Ann Arbor staging classification.

Treatment consisting of a combination chemotherapy R-CHOP scheme (Rituximab, cyclophosphamide, hydroxydaunomycin, Oncovin, and prednisone), eight cycles every three weeks, was initiated soon after the diagnosis of the NHL relapse was made. An excellent response was observed following eight cycles of R-CHOP, without any signs of relapse 20 months after the start of treatment.

## 3. Discussion

Extranodal involvement of NHL is a common finding. However only 10 to 35% of the NHL cases present themselves with a primary extranodal NHL. The most common site of extranodal involvement of NHL is the gastrointestinal tract and skin. Female genital tract involvement can in rare cases be a site of origin [2].

Freeman et al. reported in 1972 a case series of 1,467 extranodular lymphomas, of which only 3 (0.2%) were found to originate in the cervix [2]. A publication in 1995 states that 18 (0.6%) out of 2,733 NHL cases originated from the uterus, cervix, or paracolpos [3]. Lymphoma only accounts for 0.3% of all cervical cancers, making it a rare malignancy. In recent decades, the incidence of extranodal NHL has increased. The hypothesized etiology of the increase includes immunosuppressive treatments, HIV-AIDS-related immunosuppression, environmental exposure to pesticides, and improved diagnostic techniques [4].

Due to the rarity of extranodal cervical NHL, no prospective randomized trials exist to evaluate diagnostic tools or treatment options. The review stated below is thereby mostly based on case reports and small case series.

The clinical presentation of primary cervical extranodal NHL is similar to that of squamous cell carcinoma of the cervix. The most frequent encountered symptom is vaginal blood loss, which can be pre- or postmenopausal [5, 6]. In some cases asymptomatic patients have been diagnosed during routine gynecological exam, incidental after uterine surgery for unrelated reasons or rarely after routine cervical cytology smear [7]. In contrast to patients with nodal NHL, patients with cervical extranodal NHL often lack B symptoms (fever, weight loss, fatigue, and night sweats), thus giving a low rate of suspicion [8]. The median age at diagnosis of cervical NHL is 44 years, ranging from 27 to 80 years [6].

Physical examination often reveals a diffusely enlarged or bulky, sometimes barrel-shaped, cervix, often with absence of erosive or exophytic structures. Therefore, they are clinically difficult to differentiate from other benign lesions, like cervical fibroids, cervical inflammation, or nabothian cysts.

As encountered in our case, the Papanicolaou cervical smear (cytology) is only of limited use in the diagnosis and screening of cervical lymphomas. Most lymphomas arise from the cervical stroma rather than the mucosa, thus usually lacking surface ulceration and abnormal cervical cytology. An abnormal cervical cytology smear has been reported in 20%–50% of the cases, and only 6.5% are reported to have abnormal lymphoid cells [5, 8–10]. Cervical biopsies with a hematoxylin and eosin staining are therefore obligatory in the diagnosis of cervical lymphomas. However, in some cases a deep incisional or excisional biopsy and immunohistochemical stains, listed in Table 1, are necessary to establish a definite diagnosis when initial biopsy and stains are nonconclusive [5].

Histologically, cervical NHL appears to be similar to NHL arising in other sites. The majority of uterine cervix lymphomas are diffuse large B-cell type, most frequently being high grade with positive CD-20 staining [7].

Primary extranodal cervical NHL has a rapid growth pattern. Chan et al. [5] reviewed a series of 6 cases which all presented with a cervical lesion 6 cm or greater with a normal pelvic examination within the past year. Muntz et al. [6] reviewed a series of cervical NHL stage IE in which half of the patients had a tumor size more than 4 cm at presentation.

Pelvic ultrasound and CT scanning are useful in the diagnosis and staging of cervical malignancies. However, MRI is the most effective method for imaging evaluation of the cervix. MRI could possibly be used to differentiate cervical and uterine lymphomas from other entities. Cervical NHL is best defined on T2-weighted images or contrast-enhanced T1-weighted images [13, 14]. Suggestive MRI findings for lymphomas include an isointense or hypointense signal relative to the myometrium on T1-weighted images, a relatively homogenous high-intensity signaling on T2-weighted images, and lack of clear margination with moderate uniform enhancement [13, 15]. In contrast to previous reports, in our case the tumor presents itself with a heterogenous high-intensity signaling on T2-weighted images. FDG-PET imaging is increasingly being used and found to be useful in the initial staging, treatment monitoring, and followup in extranodal cervical NHL [16].

All NHL, including extranodal cervical NHL, are staged following the Ann Arbor staging classification [17]. Extranodal NHL is staged as stage IE, IIE, IIIE, or IVE, stage IE involving only one extralymphatic organ or site, stage IIE as a localized involvement of an extralymphatic organ or site and involvement of one or more lymph node regions on the same side of the diaphragm, stage IIIE as a localized involvement of extralymphatic organ or site and involvement of one or more lymph node regions on both sides of the diaphragm, and stage IVE as diffuse or disseminated involvement of one or more extralymphatic organs or sites with or without lymph node involvement. However, the additional use of the FIGO classification does give useful information concerning the disease bulk.

Because of its rarity and lack of prospective trails, no consensus concerning the optimum management of primary cervical extranodal NHL has been defined in the literature. Treatment regimens of primary cervical extranodal NHL have been reported in several case reports and case series which included surgery, chemotherapy, radiotherapy, or a combination of two or three treatments.

Chemotherapy combined with or without radiation therapy is considered to be superior compared to surgery in all but the smallest cervical NHL [3].

This statement is based on excellent responsiveness of NHL to combined chemoradiotherapy and reports where a hysterectomy after chemotherapy often shows a lack of detectable residual tumor [18, 19]. The 5-year survival rate of combined chemoradiotherapy in extranodal NHL is reported to be 80%, a 20–30% improvement on the treatment with radiotherapy alone [20].

Standard chemotherapy consists of a combination of cyclophosphamide, hydroxydaunomycin, Oncovin, and prednisone (CHOP) combined with Rituximab (R-CHOP), a monoclonal antibody directed against the CD-20 antigen. The addition of Rituximab in diffuse large B-cell NHL over eight cycles has yielded superior result compared to CHOP solely (75% compared with 63%, *P* = .005), thus becoming the standard treatment regime [21]. Treatment regimens consist of a minimum of 3 cycles or 8 cycles in bulky disease. R-CHOP, compared to other chemotherapy protocols, is associated with a low rate of infertility, and thus a sole treatment without radiotherapy (even in bulky disease) should be considered in young patients who desire fertility preservation [8]. It should be noted that ovarian translocation out of radiation field could also be a potential way to preserve ovarian function in cases where radiation therapy is considered.

In the past bulky stage IE disease was treated aggressively. Some authors advised combining high-dose radiation therapy with brachytherapy to control central tumor burden [6] and others a combination CHOP chemotherapy with radiation therapy [3]. Recently however an increasing amount of reports state that excellent result may be obtained with a treatment of R-CHOP 6–8 cycles alone [18].

In more advanced stage IIE to IVE bulky disease, chemotherapy solely may not be sufficient.

In these cases with a bulky pelvic tumor a combination of chemotherapy followed by radiation therapy is occasionally recommended [3, 8]. However the role of radiation therapy remains inconclusive because of the lack of prospective studies since the Rituximab era [22].

Patients with extranodal NHL tend to have a worse prognosis compared to patients with nodal NHL. This is mainly caused by inaccurate or delayed diagnosis and thus treatment. However, the prognosis may be excellent, compared to squamous cell carcinoma, if the cervical extranodal NHL is diagnosed and treated correctly in an early stage. The 5-year survival rates have been reported to be 89% in these early stage (IE-IIE) cases [8].

Cervical extranodal NHL is an uncommon disease that provides clinicians with a diagnostic challenge. The diagnosis is difficult to be made and is often delayed because of its rarity, its low rate of suspicious findings, and the normal cervical smear. It is critical that clinicians, radiologists, and pathologists have awareness for this rare disease.

If diagnosed and treated without delay and in an early stage the prognosis of cervical extranodal NHL is significantly better compared with the prognoses of the more common squamous cell carcinomas of the cervix, thus making a correct and swift diagnosis crucial.

## Figures and Tables

**Table 1:** Interpretation of immunohistochemical stains in large lymphoid cells lymphomas [11, 12].

Immunohistochemical stain	Interpretation
CD45+	Marker for leukocyte common antigen found in hematopoietic cells, thus indicating lymphoma
S100+, PNL2+	Marker for melanoma
Cytokeratin+	Marker for carcinoma
CD20+	Marker for B-cell lymphoma
CD10+, Bcl2−, Ki67 >99%	Favours Burkitt's lymphoma
CD10+/−, Bcl2+	Favours diffuse large B-cell lymphoma
CD3+, CD20−	Marker for T-cell lymphoma
